# Spatial scaffolds or spatial dependency? A structured evidence map of AR/MR interfaces for piano learning and practice

**DOI:** 10.3389/fpsyg.2026.1842929

**Published:** 2026-09-15

**Authors:** Yueyun Wang, Qiuyan Wei

**Affiliations:** 1 Herzen University Russia, Saint Petersburg, Russia; 2 Saint Petersburg State Institute of Culture, Saint Petersburg, Russia

**Keywords:** augmented reality, cognitive load, mixed reality, notation transfer, piano learning, sight-reading, spatial computing

## Abstract

Augmented- and mixed-reality (AR/MR) systems for piano learning and practice provide spatially aligned key highlights, piano-roll cues, virtual hands, performance feedback, and gamified guidance. Previous reviews have already identified unresolved questions concerning sight-reading, cognitive load, retention, transfer, and the possibility of interface dependence. The present review therefore does not treat these issues as previously unrecognized research gaps. Instead, it provides a structured evidence map of empirical studies of AR/MR and spatially augmented interfaces for piano learning and practice and examines how interface design relates to the kinds of learning outcomes that have been measured. Backward and forward citation tracking, supplemented by open bibliographic metadata and publisher searches, identified 36 candidate records. After two duplicate, expanded, or preliminary reports were removed or merged, 34 records were screened, 20 reports underwent detailed eligibility assessment, and 18 empirical reports were retained in the core evidence map. The reports were coded according to two dimensions: notation continuity—notation-preserving, notation-bridging, and notation-replacing interfaces—and scaffold withdrawal—persistent, contingent, progressively faded, or removed guidance. The evidence is strongest for proximal outcomes, including immediate note accuracy, task completion, usability, engagement, motivation, and perceived workload. A smaller number of studies examined repeated practice, motor-skill development, or performance after guidance was reduced or removed. However, delayed unaided transfer to conventional staff notation remains weakly tested. The available evidence therefore indicates that spatial guidance can provide effective immediate task support but does not establish that it consistently produces independent music-reading skills or that it necessarily creates dependency. The review's contribution lies in linking notation continuity, scaffold withdrawal, and outcome distance to clarify what existing studies can—and cannot—demonstrate about learning.

## 1 Introduction

### 1.1 Background: spatial computing in piano learning and practice

The commercialization of spatial-computing devices has increased interest in AR/MR music-learning environments that combine physical keys, spatial cues, and immediate feedback. Commercial spatial piano-learning applications have extended these interfaces beyond laboratory prototypes and into individual, classroom, and teacher-supported settings (Amm et al., 2024; Wilson and Pfeiffer, 2023).

### 1.2 Academic problem: what do AR interfaces change?

The critical issue is not whether AR improves general efficiency, but which specific cognitive links it alters. First, spatial overlays might reduce visual search and split-attention costs between scores and keyboards. Second, this design may primarily decrease extraneous cognitive load rather than facilitate musical symbol processing (Sweller, 1988). Third, piano sight-reading relies heavily on note decoding, look-ahead reading, and spatial familiarity (Imai-Matsumura and Mutou, 2023). Thus, it remains an empirical question whether AR cues support actual note-to-key mapping or replace score-driven learning with perception-action tracking. Because prior reviews have already raised concerns about the distinction between guided execution and independent music reading, the remaining analytical task is not simply to restate this gap, but to determine how existing empirical systems preserve, bridge, or replace notation and whether their guidance is maintained, reduced, or removed during assessment.

### 1.3 Positioning the review in relation to prior work

The present review builds on, rather than replaces, previous classifications of augmented piano-learning systems. Deja et al. (2022) organized augmented piano prototypes around learner-oriented themes such as sight-reading, posture and movement, motivation, and improvisation. Wilson and Pfeiffer (2023) focused primarily on feedback modalities in augmented- and virtual-reality piano tutors, whereas Amm et al. (2024) examined mixed-reality strategies including traditional and alternative notation, virtual hands, key highlighting, theory instruction, and remote teaching. These reviews have already questioned whether augmented interfaces improve transferable musical skills or primarily facilitate performance within the assisted environment.

However, existing studies have not been systematically mapped according to notation continuity, scaffold withdrawal, and outcome distance. To organize this evidence, the present review uses three linked dimensions. Notation continuity captures whether conventional notation is preserved, explicitly bridged to spatial cues, or replaced by direct action guidance; scaffold withdrawal captures whether guidance remains persistent, becomes contingent, is progressively faded, or is removed; and outcome distance distinguishes proximal assisted performance from intermediate learning and distal unaided retention or transfer. Together, these dimensions clarify whether the cognitive processes supported during training remain aligned with those required when assistance is reduced or removed.

### 1.4 Review aim, questions, and contributions

This structured evidence map addresses three questions. First, how do empirical AR/MR and spatially augmented interfaces for piano learning and practice differ in the extent to which they preserve, bridge, or replace conventional notation? Second, do the reported outcomes primarily concern proximal assisted performance, intermediate learning processes, or distal unaided retention and transfer? Third, how is spatial guidance managed across training and assessment, and what evidence is available to distinguish temporary scaffolding from persistent cue support?

The review makes three corresponding contributions. First, it separates direct empirical evidence on piano learning and practice from contextual evidence derived from prior reviews, broader AR education, cognitive-load research, music sight-reading, gamification, and XR accessibility. Second, it applies a notation-continuity classification systematically across the included empirical studies rather than presenting notation-preserving, notation-bridging, and notation-replacing interfaces only as conceptual categories. Third, it extends this classification by coding scaffold withdrawal as persistent, contingent, progressively faded, or removed and relates these conditions to the distance of the reported outcome.

The review's novelty therefore lies in operationalizing these established concerns as a framework linking interface design, assessment conditions, and outcome distance.

## 2 Review method

### 2.1 Review design

This article adopts a structured evidence-mapping approach rather than a systematic review claiming exhaustive database coverage. The purpose of the review was to identify and comparatively map empirical studies of augmented-reality, mixed-reality, and spatially augmented interfaces used for piano or keyboard learning. Particular attention was given to the role of conventional notation, the persistence or withdrawal of instructional guidance, and the distance between the assisted training task and the reported learning outcome.

The evidence-identification procedure was completed on 17 July 2026. The prespecified publication eligibility window covered studies published from 1 January 2011 through 31 March 2026. Records first published after 31 March 2026 were noted during searching but were outside the eligibility window for the core evidence map. The review included English-language journal articles, full conference papers, and proceedings papers reporting original empirical findings. Because the included studies varied substantially in technology, instructional task, participant expertise, study design, duration, and outcome measurement, the review used narrative evidence mapping rather than quantitative meta-analysis.

### 2.2 Evidence sources and identification strategy

Candidate studies were identified through backward and forward citation tracking from five review-oriented and empirical publications that collectively represented major strands of the augmented-piano literature, including feedback modality, instructional strategy, notation design, passthrough MR, and improvisation. The seed set was selected to maximize conceptual and technological coverage rather than to represent independent research groups. Citation tracking was iterative, with newly identified eligible reports also checked for relevant references and citing publications. To validate coverage, targeted searches of Association for Computing Machinery (ACM) Conference on Human Factors in Computing Systems (CHI), Institute of Electrical and Electronics Engineers (IEEE) International Symposium on Mixed and Augmented Reality (ISMAR), and IEEE Conference on Virtual Reality and 3D User Interfaces (VR) proceedings were additionally conducted using combinations of piano/keyboard, AR/MR/VR/XR, and learning/training/practice terms. No additional eligible full empirical report published within the prespecified publication window was identified.

Citation tracking was supplemented by targeted searches of publisher webpages, DOI records, and open bibliographic sources, including Crossref, dblp computer science bibliography (dblp), Education Resources Information Center (ERIC), and other accessible scholarly metadata services. Search terms were organized into three concept clusters:

1. technology terms, including “augmented reality,” “mixed reality,” “virtual reality,” “extended reality,” “spatial computing,” “spatial augmentation,” “projected augmentation,” and “augmented piano”;
2. domain terms, including “piano,” “keyboard,” “piano learning,” “piano tutor,” “piano practice,” “music reading,” and “sight-reading”; and
3. outcome terms, including “learning,” “training,” “performance,” “accuracy,” “cognitive load,” “workload,” “retention,” “transfer,” “usability,” “engagement,” “motivation,” and “dependency.”

A representative search combination was:

(“augmented reality” OR “mixed reality” OR “virtual reality” OR “spatial computing” OR “spatial augmentation” OR “projected augmentation” OR “augmented piano”)

AND

(piano OR keyboard OR “piano learning” OR “piano tutor” OR “piano practice” OR sight-reading)

AND

(learning OR training OR practice OR performance OR accuracy OR “cognitive load” OR workload OR retention OR transfer OR usability OR engagement).

### 2.3 Eligibility criteria

Studies were included in the core empirical evidence set when they met all of the following criteria:

1. the publication reported original empirical data;
2. the study involved human participants;
3. the learning or performance task involved a piano, electronic keyboard, or directly related piano-learning activity;
4. the instructional system used AR, MR, an optical or video see-through display, a virtual overlay aligned with a physical piano, or spatial projection directly coupled to the keyboard; and
5. the study reported at least one learning-related, performance-related, cognitive, or user-experience outcome.

Eligible outcomes included note and rhythm accuracy, task-completion time, music-reading performance, music literacy, fingering, hand posture, motor technique, improvisation, cognitive load, workload, usability, engagement, motivation, retention, transfer, and performance after guidance reduction or removal.

Studies were excluded when they:

1. described only the technical development of a system without learner or user data;
2. did not involve piano or keyboard learning;
3. focused on automatic piano performance, robotic playing, gesture recognition, or dataset construction without an instructional evaluation;
4. did not report a learning-, performance-, workload-, or user-experience-related outcome;
5. were reviews, commentaries, editorials, demonstrations, proposals, abstracts, or commercial product descriptions rather than full empirical reports;
6. fell outside the date or language limits;
7. did not provide sufficient information for an eligibility decision; or
8. duplicated, preceded, or was subsequently expanded into a more complete empirical publication.

### 2.4 Study selection and deduplication

The evidence-identification process yielded 36 candidate records. Two preliminary or expanded records were consolidated with fuller publications: the P.I.A.N.O. demonstration was merged with Rogers et al. (2014), and the ImproVISe preprint was consolidated with Deja et al. (2025).

After this process, 34 unique titles and abstracts were screened. Fourteen records were excluded during title and abstract screening because they were reviews or commentaries, technical demonstrations, extended abstracts, publications outside the date limits, or otherwise inconsistent with the eligibility criteria. Twenty reports proceeded to detailed eligibility assessment. Two reports were excluded at this stage: one did not contain eligible human learner data, and sufficient information could not be obtained for the other. The final evidence map therefore comprised 18 empirical reports. The evidence-identification and study-selection process is summarized in Table 1.

**Table 1:** Evidence-identification and study-selection flow.

Selection stage	Number of records
Candidate records identified through citation tracking and open-metadata searching	36
Duplicate, expanded, or preliminary reports removed or merged	2
Unique records remaining after deduplication and merging	34
Titles and abstracts screened	34
Records excluded after title and abstract screening	14
Full-text or detailed reports assessed for eligibility	20
Full-text reports excluded	2
Core empirical reports included in the evidence map	18

### 2.5 Data extraction

For each included report, we extracted publication details, participant characteristics, study design and duration, system and hardware information, instructional task and comparison condition, outcomes and findings, notation relationship, scaffold-withdrawal condition, and evidence of retention, no-guidance performance, or transfer. Details not reported in the primary publication were coded as NR rather than inferred from secondary sources.

### 2.6 Interface and outcome coding

The included systems were coded according to two main design dimensions.

The first dimension was notation continuity. Notation-preserving interfaces retained conventional staff notation as the primary information source and used spatial technology mainly for localized feedback, hand-position guidance, rhythm support, fingering, or error correction. Notation-bridging interfaces explicitly connected conventional notation with spatial or keyboard-based cues. Notation-replacing interfaces allowed the task to be completed mainly through falling notes, piano-roll bars, direct key highlighting, virtual hands, or other action cues without requiring conventional notation decoding.

The second dimension was scaffold withdrawal. Persistent guidance remained continuously available during practice and assessment. Contingent or adaptive guidance appeared only after an error, hesitation, or learner request. Progressively faded guidance was systematically reduced across trials or sessions. Removed guidance was unavailable or substantially reduced during a subsequent assessment.

Systems were coded at the level of the specific instructional mode rather than automatically assigning one category to the entire application. This approach allowed hybrid systems to be coded differently when they combined staff notation, piano-roll guidance, virtual hands, optional hints, or no-guidance assessment modes.

Outcomes were additionally classified according to their distance from the assisted learning condition. Proximal outcomes were measured while the same interface guidance remained present, including immediate accuracy, task completion, workload, usability, and engagement. Intermediate outcomes included change across repeated practice sessions, motor-skill development, strategy use, or performance after partial guidance reduction. Distal outcomes required delayed performance, a novel task, conventional staff notation, or complete removal of the trained interface.

### 2.7 Synthesis approach

Because of substantial methodological heterogeneity, no pooled effect size was calculated. The reports were synthesized narratively and organized according to interface type, scaffold-withdrawal condition, and outcome distance. Direct empirical piano-learning evidence was distinguished from contextual sources, including prior reviews, broader AR cognitive-load research, music sight-reading studies, game-based music-learning research, and XR accessibility literature.

## 3 Evidence map of the included studies

### 3.1 Characteristics of the included studies

The final evidence map comprised 18 empirical reports published between 2013 and 2025. The reports differed substantially in participant expertise, sample size, learning task, technological platform, study duration, comparison condition, and outcome measurement. Some reports evaluated immediate note accuracy, rhythm, task completion, workload, usability, or motivation under continued interface guidance. Others examined broader outcomes, including music literacy, motor technique, hand posture, improvisation, or performance after guidance was reduced or removed.

To make the basis of the synthesis transparent, Table 2 summarizes the participant samples, research designs, systems, comparison conditions, outcomes, and principal findings of the included studies. Table 3 then maps the same reports according to notation continuity, scaffold withdrawal, and the presence or absence of retention or transfer assessment.

**Table 2:** Characteristics and main findings of the included empirical reports.

References	Participants	Design and duration	System and comparison	Main outcomes	Main findings
Chow et al. (2013)	Seven student participants with piano experience ranging from none to advanced	Informal evaluation of a beginner piano-practice system	Trivisio ARvision-3D head-mounted display, marker tracking, and Musical Instrument Digital Interface (MIDI) input; no clear comparison condition	Practice feedback, perceived helpfulness, pressure, and learning beliefs	Participants considered the system helpful and believed that it could support learning, although the interface initially increased perceived pressure.
Rogers et al. (2014)	Study 1: 56 novice learners; Study 2: 18 novice learners	Comparative laboratory study followed by approximately 1 week of practice	P.I.A.N.O. projected interface compared with Finale sheet music and a Synthesia-style screen-based piano roll	Correct, missed, and incorrect notes; task duration; cognitive load; user experience; perceived musical quality	The projected interface supported faster initial learning, lower reported cognitive load, more favorable user experience, and higher perceived musical quality than the comparison conditions, although some error measures varied by condition.
Xiao et al. (2016)	Eight children aged 7–13 years	Two preliminary studies involving lesson and practice-session comparisons	Andantino projected animated characters and falling-block guidance compared with score-only practice	Practice behavior, score use, listening, memorization, engagement, and teacher observations	Children showed high engagement and often relied less on the printed score. The interface was considered useful for memorization, but formal retention and transfer were not tested.
Zeng et al. (2019)	Two adult novice participants forming one paired-play dyad	Within-pair comparison of immediate-note cues and upcoming-note hints during paired play	FunPianoAR using Epson Moverio BT-300 smart glasses; upcoming-note hints compared with immediate cues only	Note accuracy and timing differences between paired players	Guidance that included upcoming-note information produced better performance than cues that appeared only at the required moment.
Molloy et al. (2019)	NR	Short-term comparative evaluation	Gamified Mixed Reality Piano Tutor using HTC Vive, ZED Mini camera, and MIDI keyboard compared with Synthesia	Note accuracy, usability, interest, and enjoyment	Users achieved higher immediate note accuracy than with Synthesia and described the MR system as intuitive, interesting, and enjoyable.
Rigby et al. (2020)	Twenty-two participants	Short-term AR piano-tutor evaluation, including pre- and post-use measures	piARno using an HTC Vive and stereo camera; baseline or pretest comparison	Note-name recall and correct note execution	Participants improved in recalling note names and executing the corresponding notes after using the system.
Molero et al. (2021)	Five teachers and 13 students	Evaluation in an educational setting	HoloMusic XP using mixed reality and gamified piano-learning activities; no separate control group	Usability, motivation, satisfaction, and understanding of multimedia representations	Teachers and students reported favorable usability and satisfaction, increased motivation, and interest in incorporating the system into lessons.
Fang et al. (2022)	Sixteen adults aged 18–56 years; 13 reported no previous piano experience	Between-subjects comparison of visual MR and haptic learning; 30-min training with pre- and post-tests	Mixed-reality piano-learning system with on-skin haptic actuators; Visual falling-cue condition compared with an on-skin haptic-cue condition	Learning rate and user evaluation	The findings suggested that haptic actuators could supplement spatial guidance and contribute to an engaging piano-learning environment.
Cui (2023)	Forty-seven novice university piano students	Course-based intervention conducted from January to July 2021	Mobile and AR applications, including AR Pianist and Music Everywhere; no separate non-AR control reported	Self-reported progress in terminology, score reading, independent practice, performance, and technical skill	A high proportion of students reported improvement in musical terminology, score reading, independent work, performance, and technical skills. The reliance on subjective outcomes limits causal interpretation.
Chen (2023)	One hundred college students	Quasi-experimental comparison of experimental and control groups	HoloKeys AR/IoT-assisted piano course compared with conventional piano instruction	Musical literacy, resonance, rhythm, coherence, tempo, fingering, two-hand coordination, and legato performance	Students in the AR/IoT condition achieved significantly stronger course outcomes than the conventional-instruction group across several musical-literacy and piano-skill indicators.
Liu et al. (2023)	Twelve professional pianists	Comparative user study	PianoSyncAR superimposed synchronized teacher-hand discrepancies over the learner's hands and was compared with a conventional screen- or tablet-based presentation	Perceived intuitiveness, operability, usefulness, learning effectiveness, and hand-posture guidance	AR visualization was rated as more intuitive, operable, helpful, and effective than the screen-based condition for identifying and correcting hand-posture differences.
Lange-Nawka et al. (2023)	NR	Comparative user study	AR piano-roll tutor with vs. without auditory temporal hints or virtual metronome support	Piano performance, rhythm errors, cognitive load, and preference	No uniform effect emerged across the complete sample, but learners with some piano experience showed indications of fewer rhythm errors and lower cognitive load with auditory timing support.
Banquiero et al. (2023)	Thirty-three piano students	Within-study comparison of Wireframe and Solid grayscale-passthrough visualization modes	Oculus Quest 2 grayscale-passthrough MR piano system; wireframe vs. solid visualization modes	User experience and perceptions of passthrough-display limitations	Participants noticed limitations associated with grayscale passthrough, although both visualization modes produced generally satisfactory user-experience evaluations.
Banquiero et al. (2024)	Thirty-three piano students	Comparative study	Meta Quest Pro color-passthrough MR piano-learning application compared with Synthesia on an iPad and MIDI keyboard	Performance, subjective experience, presence, adverse effects, and preference	Participants performed the pieces more successfully in the MR condition and generally preferred it to Synthesia; both systems nevertheless provided effective immediate task support.
Amm et al. (2024)	Study 1: 15 participants; Study 2: 10 participants; the first sample also included three music teachers	Two user studies evaluating a theory-learning hub and a remote-lesson component	Meta Quest 2/Pro MR environment connected to a Yamaha P-45 keyboard; mainly within-participant pre/post comparisons	Correct notes and chords, confidence, usability, motivation, and co-presence	Beginners and intermediate learners improved between pre- and post-assessment and reported favorable motivation, usability, visualization, and co-presence. Participants did not regard the system as a complete substitute for a teacher.
Lu (2025)	Two hundred and ninety-seven third-year university students	Experimental and control-group training study	AR Piano application using three-dimensional visualization compared with a control condition	Piano Proficiency Test-2, left-hand motor skill, and piano-training performance	Students in the AR condition showed significantly greater post-training improvement in left-hand piano motor skill than those in the control group.
Deja et al. (2025)	Six advanced learners	Four-day controlled mixed-method study	ImproVisAR projector-based AR piano-roll and staff-notation interface compared with traditional sheet-music instruction	Cognitive load, engagement, interaction logs, interviews, and expert-rated rhythm, flow, musicality, and overall impression	ImproVisAR extended AR piano-roll instruction beyond note-following and sight-reading to the teaching of improvisation, a complex and generative musical skill. Workload declined across training, engagement remained favorable, and expert ratings of participant recordings favored the AR condition on several improvisational dimensions. Although the small sample (*n* = 6) requires cautious interpretation, the findings provide preliminary evidence that spatial piano-roll guidance can support an improvisation-training protocol.
Pan et al. (2018)	Thirteen volunteers, including two older adults	Pilot evaluation following a practice phase; single- and paired-play tasks	Epson Moverio BT-300 AR smart glasses; single- and paired-play modes	Playing performance, system experience, and enjoyment	Participants completed both single- and paired-play tasks and generally evaluated the system favorably. The study focused on immediate assisted performance and did not assess retention or notation transfer.

AR, augmented reality; MR, mixed reality; NR, not reported. Potential participant overlap between Banquiero et al. (2023) and Banquiero et al. (2024) could not be determined from the published reports. The two publications were retained as separate reports because they evaluated different devices, visualization conditions, and comparison tasks.

**Table 3:** Mapping of interface type, scaffold withdrawal, and transfer evidence.

References	Notation relationship	Scaffold withdrawal	Retention or transfer assessment	Main evidential limitation
Chow et al. (2013)	Notation-bridging	Persistent guidance	No	Informal evaluation; sample and comparison information were limited.
Rogers et al. (2014)	Primarily notation-replacing	Persistent guidance	Repeated practice was examined, but no separate delayed or staff-notation transfer test was included	Favorable outcomes were largely measured with the trained visual representation still available.
Xiao et al. (2016)	Notation-bridging; falling-block mode was notation-replacing	Persistent guidance	No formal retention or transfer assessment	Very small child sample and preliminary observational design.
Zeng et al. (2019)	Notation-replacing	Persistent guidance	No	Immediate cue-following performance was assessed in only one paired-play dyad.
Molloy et al. (2019)	Notation-replacing	Persistent guidance	No	Short-term comparison focused on accuracy and user experience.
Rigby et al. (2020)	Notation-bridging	Persistent guidance	Immediate pre/post change only	No delayed test or novel-score transfer condition.
Molero et al. (2021)	Notation-bridging	Persistent guidance	No	Small sample and predominantly subjective outcomes without a control group.
Fang et al. (2022)	Notation-replacing	Persistent guidance	No delayed retention or transfer assessment	Small sample and short training duration; no delayed retention or notation-transfer assessment.
Cui (2023)	Hybrid notation-bridging/replacing	Persistent guidance	No	Primarily self-reported progress and no separate non-AR comparison group.
Chen (2023)	Primarily notation-replacing	Persistent guidance	No separate delayed or no-guidance transfer test	Stronger design and larger sample, but improvement was assessed within the instructional course rather than after interface withdrawal.
Liu et al. (2023)	Notation-preserving	Persistent guidance	Transfer concerned posture guidance rather than notation; no delayed retention test	Professional-pianist sample and technique-specific task limit generalization to beginner sight-reading.
Lange-Nawka et al. (2023)	Notation-replacing	Persistent guidance	No	Effects varied by previous piano experience and no delayed assessment was included.
Banquiero et al. (2023)	Notation-replacing	Persistent guidance	No	Focused primarily on passthrough visualization and user experience rather than learning transfer.
Banquiero et al. (2024)	Notation-replacing	Persistent guidance	No	Performance was measured under the same assisted interface conditions used during learning.
Amm et al. (2024)	Primarily notation-bridging	Optional or contingent hints during practice; guidance removed in parts of the assessment	Immediate no-hint or reduced-guidance assessment, but no delayed transfer test	Pre/post improvement was demonstrated, but long-term independent notation transfer was not tested.
Lu (2025)	Primarily notation-replacing	Persistent guidance	No delayed transfer test	Large sample and control group, but the outcome focused on left-hand motor skill rather than independent score reading.
Deja et al. (2025)	Notation-bridging	Reduced piano-roll guidance in the Compose-on-the-Fly (COTF) mode	Performance under reduced piano-roll guidance was assessed in COTF; no formal delayed transfer assessment was included.	The small sample (*n* = 6) limits generalizability, but the study provides preliminary evidence on the use of AR piano-roll guidance for teaching improvisation, a complex and generative musical skill. Delayed transfer was not assessed.
Pan et al. (2018)	Notation-replacing	Persistent guidance	No	Pilot evaluation with a small sample; outcomes were measured under assisted single- and paired-play conditions.

Notation-preserving interfaces retain conventional notation as the main information source. Notation-bridging interfaces connect conventional notation to spatial or keyboard cues. Notation-replacing interfaces allow the task to be performed mainly through direct visual or spatial action guidance. Coding refers to the instructional mode evaluated in each study rather than necessarily to every function of the complete system.

### 3.2 Interface classification and transfer coverage

Table 3 applies the notation-continuity and scaffold-withdrawal codes to the 18 included reports and records whether each report assessed retention, performance after guidance reduction, or transfer. Coding was conducted at the level of the evaluated instructional mode because several systems combined more than one representation or support condition.

Of the 18 reports, 10 primarily evaluated notation-replacing interfaces, five evaluated notation-bridging interfaces, one evaluated a notation-preserving interface, and two involved hybrid modes. Sixteen reports retained persistent guidance during the principal assessment. Only Amm et al. (2024) and Deja et al. (2025) incorporated conditions in which the principal instructional guidance was reduced. Amm et al. included optional or no-hint assessment components, whereas ImproVisAR reduced piano-roll guidance during Compose-on-the-Fly improvisation tasks. Rogers et al. (2014) examined repeated practice, but did not include a delayed staff-notation transfer test. Among the included reports, we identified no study that assessed delayed, unaided transfer to an unfamiliar conventional score. The evidence base was therefore concentrated on cue-dominant interfaces and proximal assisted outcomes.

## 4 Conceptual clarification and interface typology

### 4.1 Relationship to previous classifications

Previous reviews classified augmented piano systems according to pedagogical function, feedback modality, and instructional strategy (Amm et al., 2024; Deja et al., 2022; Wilson and Pfeiffer, 2023). The present framework complements these approaches by coding notation continuity, scaffold withdrawal, and outcome distance. These dimensions distinguish systems with similar instructional aims according to how much symbolic decoding they require and whether guidance remains available during assessment.

### 4.2 Interpreting notation continuity

Notation continuity captures how much the note-to-action mapping remains dependent on conventional symbolic decoding. PianoSyncAR was coded as notation-preserving; piARno, the systems examined by Amm et al. (2024), and ImproVisAR were coded as notation-bridging; and P.I.A.N.O., FunPianoAR, the Mixed Reality Piano Tutor, and the Banquiero et al. systems were primarily notation-replacing.

These categories apply to evaluated instructional modes rather than entire applications. Andantino, for example, combined written-score engagement with a falling-block mode, illustrating that one system may include both bridging and replacing functions (Xiao et al., 2016).

### 4.3 Interpreting scaffold withdrawal

Scaffold withdrawal distinguishes persistent assistance from guidance that is contingent, progressively faded, or removed during assessment. Most included reports used persistent guidance, whereas Amm et al. (2024) incorporated reduced- or no-hint assessment components and Deja et al. (2025) reduced piano-roll guidance in the Compose-on-the-Fly improvisation mode. Explicit progressive fading was rare. This distinction is essential because cue dependence cannot be inferred without a guidance-withdrawal contrast.

### 4.4 Cognitive interpretation of the framework

The notation-continuity and scaffold-withdrawal dimensions identify different distributions of cognitive activity rather than assuming that AR/MR uniformly reduces processing demands. Spatially aligned information may reduce visual search, split attention, and key-location difficulty by placing action-relevant cues close to the physical keyboard. Direct piano evidence from P.I.A.N.O. and related systems is consistent with this possibility, although the effects vary across interface and learner conditions (Rogers et al., 2014; Lange-Nawka et al., 2023).

At the same time, cue-dominant interfaces may reduce the extent to which learners must actively decode pitch, rhythm, and notation-to-key relationships. This reduction may be beneficial when the instructional aim is rapid task entry, motor orientation, memorization, or participation. It becomes more consequential when improvement in conventional sight-reading is claimed, because unaided sight-reading depends on symbolic decoding, anticipation, executive control, and eye-hand span (Imai-Matsumura and Mutou, 2023; Perra et al., 2021).

The framework therefore distinguishes between lowering the difficulty of a task and strengthening the processes required for later independent performance. Notation-preserving and notation-bridging interfaces maintain greater continuity with symbolic processing, whereas notation-replacing interfaces may shift processing toward visual tracking and perception-action coupling. Persistent guidance may maintain these altered processing conditions throughout assessment, whereas contingent, faded, or removed guidance provides stronger opportunities to test whether mappings have been internalized.

These interpretations remain theoretical where component-specific cognitive measures are unavailable. The current evidence supports the possibility of load redistribution, but it does not demonstrate that all notation-replacing interfaces weaken symbolic processing or that notation-preserving interfaces necessarily produce stronger transfer. Accordingly, the framework implies an interaction between notation continuity and scaffold withdrawal: differences in symbolic learning should become most visible when guidance is reduced or removed and the assessment requires conventional notation.

### 4.5 Learner, task, and context moderators

The educational significance of notation continuity and scaffold withdrawal depends on the learner, task, learning context, and instructional goal. Beginners may benefit more from direct key-location support because they have not yet developed stable note-to-key mappings. More experienced pianists may require less locational guidance but may benefit from timing, interpretation, posture, or movement feedback. The experience-dependent findings reported by Lange-Nawka et al. (2023) illustrate that the same feedback modality may have different cognitive and performance effects across expertise levels.

Age and learning context also matter. Projected characters and falling-block guidance may support engagement and memorization among children (Xiao et al., 2016), whereas professional pianists may use augmented overlays to identify fine-grained hand-posture discrepancies (Liu et al., 2023). A notation-replacing system may therefore be appropriate for one instructional goal but poorly aligned with another.

Consequently, the framework should not be used to rank preserving, bridging, and replacing interfaces on a single continuum from good to bad. Its purpose is to evaluate whether the chosen representation, level of assistance, assessment condition, and claimed learning outcome are appropriately aligned.

## 5 Critical synthesis of the evidence

Building on the evidence map presented in Section 3, this section evaluates what the included studies collectively demonstrate about immediate task support, cognitive load, notation-related learning, and possible cue dependence. The synthesis distinguishes findings obtained while interface guidance remained available from evidence of learning after guidance was reduced or removed. This distinction is necessary because favorable performance within an assisted environment does not, by itself, establish retention, transfer, or independent music-reading ability.

### 5.1 Proximal performance, usability, and practice engagement

Across the 18 included reports, the strongest and most consistent findings concerned proximal outcomes measured while learners were using the same interface guidance provided during practice. Projected piano rolls, upcoming-note cues, key highlighting, virtual hands, and spatially aligned feedback often supported immediate note selection, timing, task completion, or motor execution. For example, Rogers et al. (2014) found that the projected P.I.A.N.O. interface supported faster initial learning and more favorable workload and user-experience outcomes than screen-based piano-roll and conventional notation conditions. Zeng et al. (2019) reported better performance when AR guidance included information about upcoming notes, while Molloy et al. (2019) found higher immediate note accuracy and favorable user evaluations for a gamified mixed-reality tutor compared with Synthesia. Banquiero et al. (2024) similarly reported stronger performance and subjective experience in a color-passthrough MR condition than in a Synthesia condition.

Evidence also extends beyond small prototype evaluations. Chen (2023) reported stronger musical-literacy and piano-skill outcomes among 100 college students receiving AR/IoT-supported instruction than among students in a conventional teaching condition. Lu (2025) likewise found greater improvement in left-hand motor performance among 297 university students receiving AR-supported training. These studies indicate that spatial technologies can contribute to broader instructional and motor outcomes when embedded in structured training. However, neither study isolated delayed unaided transfer to conventional staff-based sight-reading.

ImproVisAR is particularly distinctive within this evidence base because it extends spatial piano-roll guidance from the reproduction of predetermined note sequences to improvisation, where learners must generate musical responses in real time. In the four-day study, expert ratings of participant recordings generally favored the AR condition in dimensions such as rhythm, flow, musicality, and overall impression, suggesting potential benefits of the training protocol despite the very small sample (Deja et al., 2025).

A second recurring pattern concerned motivation, usability, and practice engagement. Children using Andantino showed high engagement with projected characters and falling-block guidance (Xiao et al., 2016), while students and teachers evaluating HoloMusic XP reported favorable motivation, usability, and willingness to use the system during lessons (Molero et al., 2021). Cui (2023) reported perceived improvement in terminology, score reading, independent practice, and technical skill during a course using mobile and AR applications, although these outcomes were primarily self-reported. Amm et al. (2024) also found favorable usability, confidence, motivation, and co-presence outcomes in theory-learning and remote-lesson applications.

These findings support AR/MR as a means of facilitating practice initiation, reducing early task barriers, and improving the immediate learning experience. Gamified feedback may strengthen interest and persistence, particularly for beginners who would otherwise struggle to begin or sustain practice. Nevertheless, engagement and assisted accuracy should not be treated as interchangeable with independent learning. Most favorable outcomes in this evidence set were measured while spatial cues, feedback, or augmented representations remained available. They therefore demonstrate effective task support more directly than durable musical competence after system withdrawal.

### 5.2 Cognitive load: reduction or redistribution?

Cognitive load is one of the most theoretically important but inconsistently measured outcomes in AR/MR piano learning. Direct piano evidence indicates that spatial guidance can reduce some forms of task demand, but the available findings do not support a universal load-reduction claim. Rogers et al. (2014) reported lower cognitive-load ratings for the projected P.I.A.N.O. interface than for conventional notation and a screen-based piano-roll condition. Lange-Nawka et al. (2023) found no uniform advantage of auditory temporal hints across the complete sample, although participants with some prior piano experience showed indications of fewer rhythm errors and lower cognitive load. In the four-day ImproVisAR study, workload ratings declined across training sessions (Deja et al., 2025).

These findings are consistent with broader AR research showing that cognitive-load effects depend on interface design, feedback modality, learner expertise, and task complexity rather than on the presence of AR itself (Buchner et al., 2022; Zhu et al., 2026). Spatially aligned cues may reduce visual search, split attention, and key-location demands by placing action-relevant information close to the keyboard. At the same time, they may introduce or alter other demands, including monitoring dynamic overlays, coordinating visual cues with motor action, anticipating cue timing, and switching between augmented information and conventional notation.

The available evidence is therefore better interpreted in terms of load redistribution than universal load reduction. However, current piano studies have not directly isolated all of these components. Visual-search load, interface-management load, visuomotor-mapping load, rhythm-anticipation load, and symbolic-decoding load should be treated as analytically distinct rather than assumed to be reflected in a single overall workload score. A decrease in total reported workload does not necessarily mean that music-reading processes have become more efficient; it may also indicate that the interface has reduced or temporarily replaced the need for symbolic decoding.

### 5.3 From assisted execution to independent notation transfer

The central educational issue is not whether learners can perform successfully while augmented cues remain available, but whether the knowledge and skills developed during practice remain usable when those cues are reduced, removed, or replaced by conventional staff notation. The evidence map shows that this question has been addressed only partially.

Several studies provide stronger evidence than a single assisted performance test. Rogers et al. (2014) examined learning across repeated practice sessions rather than only one exposure. Rigby et al. (2020) reported immediate pre- to post-use improvement in note-name recall and correct note execution. Amm et al. (2024) incorporated optional hints during practice and assessed parts of performance with reduced or removed guidance. Deja et al. (2025) conducted a four-day study in which the Compose-on-the-Fly (COTF) mode reduced piano-roll guidance and required learners to generate their own improvisational material. In the Questions-and-Answers task, two bars were provided as a call followed by a two-bar learner response, while the Variations task used a 4-bar/4-bar structure. This design therefore provided a partial test of performance under reduced spatial guidance rather than simply measuring performance with the full AR scaffold continuously available. These designs move beyond simple usability evaluation and indicate that some augmented systems can support learning across time or after partial cue reduction.

Nevertheless, these outcomes do not constitute a direct test of delayed transfer to conventional sight-reading. Immediate pre-post change may reflect short-term familiarity with the trained material. Performance after partial cue removal may demonstrate reduced reliance on a specific interface without showing that learners can read a novel conventional score. Importantly, ImproVisAR was designed to teach improvisation rather than conventional sight-reading. Its COTF condition should therefore be interpreted as evidence concerning performance of an abstract, generative musical skill under reduced piano-roll guidance, rather than as a test of transfer to conventional notation. The study consequently provides relevant evidence for scaffold reduction within improvisation learning, although it does not address delayed notation transfer. None of the included studies provided a clear comparison of delayed, unaided performance on an unfamiliar conventional score following notation-preserving, notation-bridging, and notation-replacing training.

Notation-replacing interfaces are not inherently educationally inferior; they may support beginner access, memorization, motor orientation, improvisation, and engagement. However, claims about conventional music literacy require assessment from staff notation after spatial cues have been removed. Current evidence remains stronger for guided execution than for delayed, unaided transfer.

### 5.4 What current evidence does—and does not—show about dependency

The term dependency requires more stringent evidence than the observation that learners perform better when assistance is available. Three outcomes should be distinguished. Assisted performance refers to how accurately or efficiently learners perform while spatial cues remain present. Assistance dependence refers to a marked decline when those cues are withdrawn. Negative transfer requires evidence that prior AR/MR training produces poorer subsequent performance in an unaided conventional task than an appropriate comparison condition.

Most included studies directly support only the first of these outcomes. Learners often performed more accurately, reported lower workload, or expressed greater confidence and enjoyment while using augmented guidance. A small number of studies assessed performance after partial guidance reduction or across repeated sessions, providing preliminary evidence that learning can extend beyond a single assisted trial. However, the evidence map does not demonstrate that AR/MR piano training generally causes assistance dependence, and it does not show that notation-replacing practice produces negative transfer to conventional music reading.

The possibility of cue dependence remains theoretically plausible, particularly when three conditions occur together: conventional notation is replaced, spatial guidance remains continuously available, and the principal assessment reproduces the same assisted interface used during practice. Under these conditions, high performance may reflect efficient cue following without demonstrating that the learner has internalized symbolic or spatial mappings. Yet this possibility should be described as a risk or hypothesis rather than as an established empirical effect.

Accordingly, dependency should be treated as a hypothesis requiring a guidance-withdrawal contrast rather than as an established effect of AR/MR piano learning.

## 6 Limitations and boundary conditions

### 6.1 Limits of self-guided practice

AR/MR systems can provide immediate feedback during self-guided practice, but the included evidence did not evaluate whether such systems can reproduce the full diagnostic and interpretive role of a teacher. Available reports position them primarily as complementary tools for note selection, rhythm, visualization, repetition, and selected forms of hand-position feedback (Amm et al., 2024; Sellers and Waddell, 2024). Claims about replacing conventional instruction therefore remain beyond the scope of the current evidence base.

### 6.2 Access and hardware constraints

Access depends on device cost, tracking accuracy, latency, visual clarity, wearing comfort, calibration, and available practice space. Alternative notation and multimodal guidance may broaden access for some learners, but direct evidence from piano-learning studies remains limited. Accessibility claims should therefore be aligned with the needs and goals of specific learner groups rather than generalized across users (Burke et al., 2025; Olivares et al., 2025).

### 6.3 Limitations of the evidence base

Although several recent studies used larger samples or comparative designs, much of the evidence base remains short-term and methodologically heterogeneous. Outcome measures vary across performance, workload, usability, motivation, and motor skill, while delayed transfer tests remain rare. Novelty effects and assisted performance are also difficult to distinguish from durable learning. These limitations preclude quantitative aggregation and require cautious interpretation of claims about learning, transfer, and dependency (Amm et al., 2024; Buchner and Kerres, 2023).

## 7 Framework-derived research priorities

Previous studies have already recommended delayed testing, richer process measures, and greater ecological validity. The present agenda therefore focuses on predictions derived specifically from the notation-continuity and scaffold-withdrawal framework.

### 7.1 Immediate performance and notation transfer

Under matched training duration and baseline ability, the framework predicts an interaction between notation continuity and scaffold withdrawal. While guidance remains available, notation-replacing interfaces should produce higher immediate note accuracy and/or faster task completion than notation-preserving conditions, particularly for novice learners, because they reduce visual-search and symbolic-decoding demands. When learners subsequently perform an unfamiliar conventional score without guidance, notation-preserving and notation-bridging training should produce higher accuracy and/or a smaller performance decrement than notation-replacing training. This transfer difference should be strongest when guidance has remained persistent and should be attenuated when guidance has been progressively faded or removed during training. Prior notation expertise is expected to moderate this pattern, with smaller between-interface differences among more experienced pianists.

### 7.2 Persistent, contingent, and faded guidance

Independent of notation type, persistent guidance should produce a larger assisted-to-unaided performance decrement than contingent or progressively faded guidance under matched practice duration. Contingent or faded guidance should therefore produce stronger no-guidance retention or transfer, even if it does not maximize performance while assistance remains available.

### 7.3 Cognitive-load redistribution

Lower overall workload does not necessarily indicate improved music-reading efficiency. Spatial guidance may reduce visual search and key-location demands while also reducing symbolic decoding or increasing interface monitoring. Future research should combine workload ratings with process indicators such as gaze switching, hesitation, timing errors, and help requests.

### 7.4 Evidence required for dependency claims

Assistance dependence cannot be inferred simply because learners perform better while cues are available. A direct test requires guidance withdrawal, unfamiliar material, an appropriate comparison group, and preferably delayed assessment. Without such evidence, studies should report cue-supported performance or potential cue reliance rather than established dependency.

## 8 Conclusion

The available evidence supports AR/MR piano interfaces most strongly as tools for immediate task support, engagement, and guided motor performance. Some studies have examined repeated practice or performance after partial guidance reduction, but delayed unaided transfer to conventional staff notation remains weakly tested. The evidence therefore does not establish that AR/MR necessarily reduces cognitive load or creates dependency. Its educational effects depend on whether notation is preserved, bridged, or replaced, how guidance is withdrawn, and how far the assessment is removed from the assisted training condition. The proposed framework clarifies these conditions and provides a basis for testing when spatial guidance functions as a temporary scaffold rather than a persistent performance aid.
